# Occurrence of *Strongyloides stercoralis* in Yunnan Province, China, and Comparison of Diagnostic Methods

**DOI:** 10.1371/journal.pntd.0000075

**Published:** 2007-10-31

**Authors:** Peter Steinmann, Xiao-Nong Zhou, Zun-Wei Du, Jin-Yong Jiang, Li-Bo Wang, Xue-Zhong Wang, Lan-Hua Li, Hanspeter Marti, Jürg Utzinger

**Affiliations:** 1 Department of Public Health and Epidemiology, Swiss Tropical Institute, Basel, Switzerland,; 2 National Institute of Parasitic Diseases, Chinese Center for Disease Control and Prevention, Shanghai, People's Republic of China; 3 Helminthiasis Division, Yunnan Institute of Parasitic Diseases, Simao, People's Republic of China; 4 Medical and Diagnostic Services, Swiss Tropical Institute, Basel, Switzerland; Yale Child Health Research Center, United States of America

## Abstract

**Background:**

*Strongyloides stercoralis* is a neglected soil-transmitted helminth species, and there is a lack of parasitologic and epidemiologic data pertaining to this parasite in China and elsewhere. We studied the local occurrence of *S. stercoralis* in a village in Yunnan province, China, and comparatively assessed the performance of different diagnostic methods.

**Methodology/Principal Findings:**

Multiple stool samples from a random population sample were subjected to the Kato-Katz method, an ether-concentration technique, the Koga agar plate method, and the Baermann technique. Among 180 participants who submitted at least 2 stool samples, we found a *S. stercoralis* prevalence of 11.7%. Males had a significantly higher prevalence than females (18.3% *versus* 6.1%, *p* = 0.011), and infections were absent in individuals <15 years of age. Infections were only detected by the Baermann (highest sensitivity) and the Koga agar plate method, but neither with the Kato-Katz nor an ether-concentration technique. The examination of 3 stool samples rather than a single one resulted in the detection of 62% and 100% more infections when employing the Koga agar plate and the Baermann technique, respectively. The use of a mathematical model revealed a ‘true’ *S. stercoralis* prevalence in the current setting of up to 16.3%.

**Conclusions/Significance:**

We conclude that *S. stercoralis* is endemic in the southern part of Yunnan province and that differential diagnosis and integrated control of intestinal helminth infections needs more pointed emphasis in rural China.

## Author Summary

An estimated 30 million to 100 million people are infected with the parasitic worm *Strongyloides stercoralis*, the causative agent of strongyloidiasis, and yet this is a neglected tropical disease. The diagnosis of this parasite requires specialized techniques (e.g. Baermann and Koga agar plate method), but these are rarely employed in epidemiologic studies. We assessed the occurrence of *S. stercoralis* in a rural part of southern Yunnan province, China, and compared different diagnostic methods. At least two stool samples were obtained from 180 randomly selected individuals, and examined with four diagnostic approaches, including the Koga agar plate and the Baermann technique. Twenty-one individuals were infected with *S. stercoralis* (prevalence: 11.7%). Males were more often infected than females (18.3% *versus* 6.1%, *p* = 0.011). Infections were absent in children below the age of 15 years. The Baermann technique showed a higher sensitivity than the Koga agar plate method, and the examination of multiple stool samples improved the diagnostic performances of both methods. The use of a mathematical model suggested a ‘true’ *S. stercoralis* prevalence of 16.3%. There is a need to further study the epidemiology of strongyloidiasis in other parts of China, and control measures are required in settings with high prevalences as observed in this area.

## Introduction

Soil-transmitted helminthiases are caused by infections with intestinal nematodes, of which *Ascaris lumbricoides*, *Trichuris trichiura* and the hookworms (*Ancylostoma duodenale* and *Necator americanus*) are the most widespread species [1–3]. Collectively, these soil-transmitted helminths affect over 1 billion people and cause a huge public-health burden; yet, soil-transmitted helminthiases are so-called neglected tropical diseases [4]. *Strongyloides stercoralis* is another and even more neglected soil-transmitted helminth, although an estimated 30–100 million people are infected worldwide [2]. An infection with *S. stercoralis* occurs transcutaneously and can be perpetuated over long periods by autoinfection [5,6]. Clinical signs of *S. stercoralis*-infected immunocompetent people can be inconspicuous or even absent, but hyperinfection involving the gastrointestinal and pulmonary system is possible. Potentially fatal disseminated infections are seen in immunocompromised individuals, for example, as a result of immunosuppressive drugs or following human T-cell lymphotropic virus type 1 (HTLV-1) infection [6–8].

*S. stercoralis* is endemic in tropical and temperate zones but accurate information on the geographic distribution and the global burden of strongyloidiasis is lacking. An important underlying reason is that one of the most widely used diagnostic approaches in helminth epidemiology, i.e., the Kato-Katz method [9], fails to detect *S. stercoralis*. Moreover, microscopic examination of direct fecal smears, often used in endemic settings, has a low sensitivity [10,11]. More sensitive diagnostic approaches for detection of *S. stercoralis* larvae include the Koga agar plate method [12] and the Baermann technique [13]. Their sensitivity can be further increased by examining multiple stool samples [14].

In East Asia and Thailand in particular, the epidemiology of *S. stercoralis* has been studied in some detail. In different investigations carried out among schoolchildren and adults in northern and central Thailand, prevalences ranging between 2.3% and 28.9% were found [15–19]. *S. stercoralis* has also been investigated in other Asian countries, including Japan [20], but there is a paucity of epidemiologic data and comparison of different diagnostic methods from China. This can be illustrated by consulting the *PubMed* database (http://www.pubmed.gov) where the following search strategy “strongyloides OR strongyloidiasis AND China” resulted in only 6 hits; 3 case reports, 1 study on animal strongyloidiasis, 1 global review, and 1 old publication that looked at single and multiple species parasitic infections among 15,952 Chinese using direct-smear examinations [21] (accessed on 29 June 2007).

Here, we report findings from a cross-sectional parasitologic and questionnaire survey carried out in a random population sample in a rural setting of southern Yunnan province, China. We investigated the occurrence of *S. stercoralis* by screening multiple stool samples from the same individuals and comparatively assessed the performance of different diagnostic methods.

## Materials and Methods

### Study Area and Population

The study was carried out in Nongyang village, located in Menghai county, Xishuangbanna prefecture, Yunnan province, China (21.81° N latitude and 100.35° E longitude). The village was selected because (i) the hookworm prevalence in this area is known to be high (used as a proxy for the likely occurrence of *S. stercoralis*, as both species have the same way of transmission), and (ii) it is readily accessible by project car to assure a rapid transfer of stool samples to the nearby laboratory. Details of the study village and the population sample have been presented elsewhere [22]. In brief, the village is inhabited by members of the Bulang ethnic group, and is situated 20 km southwest of the town of Menghai in a hilly area at an elevation of 1350 m above sea level. The economy of the village is governed by the surrounding tea and sugar cane plantations, other sources of income than farming are not available. Pigs and poultry are the most common domestic animals, others include dogs and buffaloes. Whilst all houses have untreated tap water originating from a nearby river, there are no household-based sanitation facilities. A single community latrine serves the entire population, but it is not consistently used.

### Consent, Field and Laboratory Procedures

The village authorities were informed about the study, and a copy of the village family registry, containing basic demographic information, was obtained. According to the village family registry, there were some 150 households. Families with odd registration numbers (n = 78) were contacted in batches of 20–30 families per week, and all members were invited to participate in the survey. The aim and procedures of the study were explained, and an informed consent sheet was signed by the head of the household or a designated literate substitute. Pre-tested individual and household questionnaires were administered to obtain demographic (age, sex, education attainment), behavioral (wearing shoes, food consumption, personal hygiene, health care seeking) and occupational data, as well as information about the living conditions (household asset ownership, house type, sanitation infrastructure, domestic animals). Next, pre-labeled plastic containers for stool sample collection were handed out to all participants and their ability to recognize their names was checked. Each morning, filled containers were collected and replaced by empty ones for stool collection on the following day. This procedure was repeated with the goal to obtain 3 stool samples from each individual.

The stool samples were stored at ambient temperature and transferred to the laboratory within 2 hours post-collection. They were processed by the Kato-Katz technique [9], the Baermann method [13] and the Koga agar plate procedure [12]. In addition, one sub-sample per study participant was stored in sodium acetate-acetic acid-formaline (SAF) solution, forwarded to a reference laboratory in Switzerland, and processed there by an ether-concentration method for the examination of helminth eggs and intestinal protozoa [23]. All tests were performed according to standard operating procedures and carried out or initiated within 12 hours after sample collection.

Specifically, a single Kato-Katz thick smear was prepared from each stool sample and examined within 1 hour of preparation. Helminth eggs were counted separately to obtain parasite-specific infection intensity estimates. For the Baermann test, an apricot-sized stool sample was placed on a gauze-lined mesh in a glass funnel equipped with a rubber tube and a clamp, covered with deionised water and illuminated from below with a bulb. After 2 hours, the lowest 50 ml of the liquid were drained, centrifuged and the sediment examined under a microscope for *S. stercoralis* larvae (L1-stage). The Koga agar plates were freshly prepared once per week and kept at 4°C in humid conditions pending utilization. A hazelnut-sized stool sample was placed in the middle of the plate and the covered plates were incubated in a humid chamber for 2 days at 28°C. All plates were rinsed with 12 ml SAF solution, the eluent centrifuged and the sediment examined under a microscope. Recovered larvae were differentiated to distinguish *S. stercoralis* L3 larvae from hookworm larvae. Samples were considered positive if larval or adult *S. stercoralis* were observed.

### Statistical Analyses

Questionnaire data were entered in EpiData version 3.0 (EpiData Association; Odense, Denmark) and statistical analyses were carried out in STATA version 9.2 (StataCorp.; College Station, USA). Prevalence estimates for *S. stercoralis* according to the Koga agar plate and the Baermann methods were calculated by means of a mathematical model presented and used elsewhere [24,25]. Based on the relative frequency of single and repeated positive test results among the multiple stool samples submitted by the participants, the model extrapolates a ‘true’ prevalence and calculates additional test characteristics for a given method.

### Anthelminthic Treatment and Ethical Considerations

At completion of the study, free treatment with compound mebendazole (i.e., mebendazole 100 mg/tablet plus levamisole hydrochloride 25 mg/tablet; 2 tablets per day for 3 consecutive days) was offered to all inhabitants of the village by staff of the local parasite control station.

The institutional review boards of the National Institute for Parasitic Diseases (Shanghai, China) and the Swiss Tropical Institute (Basel, Switzerland) approved the study. As mentioned before, written informed consent was sought from household heads or appropriate literate substitutes.

## Results

### Population Sample and Study Cohort

In total, 283 individuals from 71 families participated in the survey (average family size: 4.0 people; range: 1–8). At least 1 stool sample of sufficient quantity to perform the various diagnostic tests was available from 234 individuals (82.7%). Two or 3 samples were submitted by 180 individuals (63.6%) and subsequent analyses were performed on this cohort. There were 98 females (54.4%) and the age of the participants ranged from 4 to 84 years. Among those aged 15 years and above, 92.0% were farmers, the others were students. The illiteracy rate in the same age group was 67.2%. The majority of those aged 14 years and below attended school (58.5%), whereas the remaining individuals were either pre-school children (26.8%) or had never attended school.

### Occurrence of *S. stercoralis*

Fourteen different parasite species were identified, 7 helminths and 7 intestinal protozoa. Very high prevalences of *A. lumbricoides* (93.3%), *T. trichiura* (88.9%) and hookworms (87.8%) were found. Here, we focus on the *S. stercoralis* results. Stool examination utilizing the Koga agar plate and the Baermann technique resulted in the identification of 19 and 21 *S. stercoralis* infections, respectively. As summarized in Table 1, all *S. stercoralis* infections detected by the Koga agar plate method were also diagnosed by the Baermann technique, whereas 2 infections were identified by the latter method only. Thus, the observed infection prevalence of *S. stercoralis*, according to Baermann was 11.7%. The Kato-Katz method and the ether-concentration technique on SAF-conserved stool specimens failed to identify even a single infection with *S. stercoralis*.

**Table 1:** Comparison of results obtained by the Koga agar plate and the Baermann methods for the diagnosis of *S. stercoralis* among 180 individuals with at least 2 stool samples examined in Nongyang village, Yunnan province, China.

	Baermann test		Total
	Positive	Negative	
Koga agar plate test positive	19	0	19
Koga agar plate test negative	2	159	161
Total	21^a^	159	180

a The sensitivity of the Koga agar plate method was 90.5% and that of the Baermann method was 100% if the combined results from both tests are considered as diagnostic ‘gold’ standard.

Table 2 shows that the prevalence of *S. stercoralis* was significantly higher among males than females (18.3% *versus* 6.1%, χ^2^ = 6.42, degrees of freedom (df) = 1, *p* = 0.011) and increased with age, albeit not significantly (χ^2^ = 8.70, df = 4, *p* = 0.069). No infections were found among participants <15 years, whereas the highest prevalence was recorded in those aged 15–24 years (19.6%). *S. stercoralis* infections were not found among students of any age. No additional risk factors for a *S. stercoralis* infection could be identified. Neither protective measures against infection, such as wearing shoes (odds ratio (OR) = 0.64, *p* = 0.516), nor hygiene behavior, e.g., hand washing before eating (OR = 1.03, *p* = 0.963) or after defecation (OR = 1.23, *p* = 0.671), willingness to see a doctor in case of illness (OR = 2.91, *p* = 0.310) or presence of domestic animals (e.g., dogs; OR = 1.88, *p* = 0.267) were associated with infection status.

**Table 2:** Number and percentage of study participants infected with *S. stercoralis* as determined by the combined Koga agar plate and Baermann techniques, stratified by sex, age group and occupation among 180 individuals from Nongyang village in Yunnan province, China.

Characteristics	No. of individuals examined	No. of individuals positive for *S. stercoralis*	Percent positive	χ^2^	*p* value
All individuals	180	21	11.7		
Sex					
Female	98	6	6.1		
Male	82	15	18.3	6.42	0.011
Age group (years)					
≤9	17	0	0		
10–14	20	0	0		
15–24	46	9	19.6		
25–39	53	5	9.4		
≥40	44	7	17.5	8.70	0.069
Occupation^a^					
Pre-school, student	40	0	0		
Farmer	138	19	13.8	6.17	0.013

a Only those individuals with known occupation were included (n = 178).

### Performance of Different Diagnostic Methods

Indicators of the diagnostic performance of the Koga agar plate and the Baermann methods, in relation to different sampling efforts, are presented in Table 3. The examination of 3 stool samples, rather than a single one, resulted in a significant increase in the number of infections detected by either method. The observed *S. stercoralis* prevalence increased from 7.3% to 11.7% when using the Koga agar plate method (an increase of 62%), and from 7.0% to 14.0% in the case of the Baermann method (an increase of 100%). Whilst using Koga agar plates, larvae were detected with equal frequencies in only 1, 2 or all 3 stool samples from infected individuals, the Baermann method often failed to detect larvae in multiple samples from the same person. Using the results of the Koga agar plate method and a mathematical model developed by Marti and Koella [24], we estimated a ‘true’ *S. stercoralis* prevalence of 12.3%. The corresponding value for the Baermann technique was 16.3%. The probability of correctly identifying infected individuals by analyzing single stool samples was estimated at 0.63 and 0.48 for the Koga agar plate and the Baermann technique, respectively.

**Table 3:** Identification of *S. stercoralis* larvae by the Koga agar plate and the Baermann methods in 3 different stool samples obtained from inhabitants of Nongyang village in Yunnan province, China, and ‘true’ prevalence and test characteristics according to a model developed by Marti and Koella (1993) [24].

Sampling effort	Koga agar plate method		Baermann method	
	Number	%	Number	%
3 stool samples analyzed	179	100	129	100
Cumulative result after analysis of				
1^st^ stool sample	13	7.3	9	7.0
2^nd^ stool sample	20	11.2	14	10.9
3^rd^ stool sample	21	11.7	18	14.0
Larvae recovered from				
1 stool sample	7	3.9	9	7.0
2 stool samples	7	3.9	6	4.7
3 stool samples	7	3.9	3	2.3
1, 2 or 3 stool samples	21	11.7	18	14.0
Estimated prevalence (SD)		12.3 (±5.1)		16.3 (±7.6)
Sensitivity of method (3 samples)		95.1		85.6
Sensitivity of individual test (SD)		63.4 (±13.8)		47.5 (±17.1)

SD: standard deviation.

Table 4 shows the effect of the sampling effort for multiple stool sample collection on the observed prevalence and the influence of the available stool quantity on the completeness of the diagnostic results. Three Koga agar plate tests could be performed for 70.5% of the 254 participants who submitted at least 1 sufficiently-large stool sample. The higher requirements of the Baermann method regarding the available stool quantity are reflected in the lower number of tests. Only 236 participants had at least one Baermann result, whereas 129 (54.7%) submitted 3 large enough stool samples. One *S. stercoralis* infection was identified by the Koga agar plate method among those participants who submitted stool samples of insufficient quantity to concurrently perform the Baermann test. Combined, the Koga agar plate and the Baermann technique identified 30 *S. stercoralis* infections among 254 individuals who submitted at least 1 stool sample of sufficient quantity to perform at least the Koga agar plate test, resulting in an observed prevalence of 11.8%.

**Table 4:** Effect of sampling efforts for stool collection and evaluation with the Koga agar plate and Baermann technique on the observed prevalence, total number of identified infections and the completeness of datasets.

Number of stool samples from participants	Koga agar plate method							Baermann method							*S. stercoralis* from 1–3 stool samples; Koga agar plate and/or Baermann method					
	Number of participants	1^st^ sample positive		1^st^ and/or 2^nd^ sample positive		1^st^ and/or 2^nd^ and/or 3^rd^ sample positive		Number of participants	1^st^ sample positive		1^st^ and/or 2^nd^ sample positive		1^st^ and/or 2^nd^ and/or 3^rd^ sample positive		Koga		Baermann		Koga & Baermann	
		No.	%	No.	%	No.	%		No.	%	No.	%	No.	%	No.	%	No.	%	No.	%
≥1	254	19	7.5	n.a.		n.a.		236	19	8.1	n.a.		n.a.		27^c^	10.6	29	12.3	30	11.8
≥2	215	13^a^	6.0	20	9.3	n.a.		180	11^a^	6.1	17	9.4	n.a.							
3	179	13^a^	7.3	20^b^	11.2	21	11.7	129	9^a^	7.0	14^b^	10.9	18	14.0						

a Results from 1^st^ sample only.

b Results from 1^st^ and 2^nd^ sample only.

c 26 cases jointly diagnosed by the Koga agar plate and the Baermann methods, 1 additional case for which no Baermann test could be performed.

n.a.: not applicable.

## Discussion

There is a paucity of parasitologic and epidemiologic investigations pertaining to *S. stercoralis* in China, and to our knowledge the performance of different diagnostic approaches has never been assessed in this setting. We carried out an in-depth study in a random population sample from a small village in Yunnan province in the south-western part of China. The collection of multiple stool samples and their screening by the Koga agar plate and the Baermann techniques revealed a prevalence of *S. stercoralis* of 11.7%.

It is conceivable that the observed prevalence still underestimates the ‘true’ prevalence, which is justified on the following grounds. First, in the absence of a diagnostic ‘gold’ standard, it is not possible to determine how often larvae failed to emigrate from the stool sample, or actually resided on the surface of the agar plate, but were not recovered. With regard to the Baermann technique, it is possible that some larvae had not yet reached the water, or settled to the ground of the funnel when the water was drained after 2 hours of exposure to light. Second, a recent study carried out in rural Malawi showed that a delay of 3 hours or more between evacuation of stool specimens by humans and processing/examining of stool samples in the laboratory resulted in a considerably decreased sensitivity of hookworm diagnosis [26]. Hence, there is concern that delays in stool processing might also negatively influence the sensitivity of diagnosing other helminth infections, including *S. stercoralis*. Future studies should investigate the effect of time from stool evacuation to laboratory examination with an emphasis on *S. stercoralis*. Third, a mathematical model [24] predicted a considerably higher prevalence of *S. stercoralis* when compared to the results of 3 stool specimens subjected to either the Koga agar plate or the Baermann technique. The application of other diagnostic methods, such as the charcoal coproculture method, which includes a culture step before harvesting the larvae by the Baermann method, and serology, might detect additional infections. Yet, based on our previous experience, we are confident that the approach taken in the current study (multiple stool samples and different diagnostic methods) detected *S. stercoralis* infections with a high sensitivity. Nonetheless, serological methods suitable to also identify very light infections should be used in future studies to further investigate the conspicuous absence of infections among children.

On the other hand, the collection of stool samples over several days under limited supervision by our research team bears the risk of mixing up collection containers at the household level. This would result in the attribution of samples from one infected person to different household members who might not be infected, thus inflating the prevalence. We are confident that this issue did not distort our data, as we provided detailed explanations to all study participants about the importance of stool collection using the designated containers, and checked the ability of at least one household member to recognize each name on the pre-lab containers. Moreover, the age and sex distribution of *S. stercoralis* infections matched the previously presented epidemiologic patterns from neighboring countries. The 21 infections diagnosed by the Baermann approach originated from 18 families, suggesting that mis-attribution was certainly not a major issue. We also assume that the participation of only 63.6% of the eligible villagers did not affect the representativeness of the sample since the age and sex distribution of these 180 individuals was similar to the remaining 103 people who failed to provide at least 2 stool samples of sufficient quantity.

Concerning the recovery of larvae from the agar plates, an attempt was made to first visually inspect the plate for larval tracks and characteristic signs of fungal and bacterial growth, but the high prevalence of hookworm larvae necessitated the recovery of the actual larvae for microscopic examination. In some cases signs of larval activity were noted, but no larvae could be recovered. Contrarily, it was shown that larvae can be present even if no signs of their activity can be detected on the surface of the agar plate [12].

We are not aware of previous community-based studies focusing on *S. stercoralis* in Yunnan province. The overall prevalence of *S. stercoralis* (11.7%) is similar to reports from northern Thailand [17]. Interestingly, southern Yunnan shares some eco-epidemiologic characteristics with northern Thailand, such as the climate, land use patterns and ethnic background. Moreover, in both settings, the prevalence of infection was significantly higher in males than in females [15,17], and increased with age, with the peak prevalence observed in adolescents and young adults [18]. Similar sex and age patterns were also reported from Laos [27]. However, in Laos and Thailand, infections were also found among children, whereas in the current study, infections were confined to individuals aged 15 years and above. These findings might point to age- and gender-specific occupational risk factors, e.g., different behavioral patterns related to agricultural activities. The absence of infections among children suggests that the main transmission sites are outside the core village, despite the precarious sanitary conditions with 86.5% of the participants reporting not using the single community latrine available in the entire village. Possibly as a result of the rather uniform educational, occupational and behavioral population characteristics, we were unable to identify additional risk factors for infection.

It is commonly assumed that even if multiple stool samples are available, no single diagnostic technique can detect all *S. stercoralis* infections. Different methods are therefore employed for the parasitological diagnosis of this helminth but they are often poorly standardized and their performance has rarely been assessed comparatively. In one of the few available studies that compared the diagnostic performance between the Koga agar plate and the Baermann method, the former technique was superior to the Baermann technique [28]. In the present study, however, the Baermann technique identified ‘all’ infections, whereas the Koga agar plate method failed to do so in 3 cases when considering all individuals who provided at least 1 stool sample of sufficient quantity (Table 4). Even taking into account the somewhat lower sensitivity of the Koga agar plate method, this technique still has advantages in field-based epidemiologic surveys. First, it allows the analysis of small stool samples, thereby reducing the number of participants who have to be excluded from the analysis due to insufficient amounts of stool, as was the case in the current study (note the total numbers of Koga agar plate and Baermann technique test results in Table 4). Second, the Koga agar plate technique also detects hookworm infections, thus allowing for concurrent diagnosis of both parasites [29]. Previous studies have shown that formaline-ether concentration methods were able to detect *S. stercoralis* infections, but compared to the Baermann and Koga agar plate methods, their sensitivity was considerably lower [10,16],[30]. The low sensitivity of direct fecal smears and the Kato-Katz method for diagnosis of *S. stercoralis* is also well known [11].

Over the past decades, profound demographic, ecologic and socio-economic changes have occurred across China [31,32], and the health system underwent significant reforms [33]. These changes also resulted in an increased availability and use of sophisticated medical techniques, including immunomodulatory drugs and organ transplantation. Consequently, it must be assumed that the immunocompromised population is expanding. Previous research has indicated that this population group is at high risk of severe disease when concurrently infected with *S. stercoralis*. Nevertheless, the obvious importance of *S. stercoralis* for public-health has yet to prompt new research into the epidemiology and control of this neglected helminth infection in China and elsewhere. In this connection, the importance of differential diagnosis of soil-transmitted helminth infections must be emphasized, particularly in view of the large-scale administration of albendazole and/or mebendazole that usually show good efficacy against *A. lumbricoides* and hookworms (only moderate efficacy against *T. trichiura*), but commonly fail to clear *S. stercoralis* [1]. We have launched additional studies with the objective of enhancing our understanding of the epidemiologic situation of *S. stercoralis* in adjacent parts of Yunnan province with different environmental, socio-economic and ethnic characteristics, and will also investigate current and future treatment options. Finally, we encourage other groups who focus their research on helminths, not to neglect *S. stercoralis* any longer.
